# Meteorological Register

**Published:** 1814-11

**Authors:** C. Blunt

**Affiliations:** Philosophical Instrument Maker, No. 38, Tavistock-Street, Covent-Garden


### METEOROLOGICAL REGISTER.

*From September the 25th, to October the 20th,* 1814.

Kept by C. BLUNT, Philosophical Instrument Maker, No. 38, Tavistock-Stieet, Covent-Garden.

| Moon. | Day. | Wind. | Barometrical Pressure. Max. | Min. | Mean. | Temperature. Max. | Min. | Mean. | |
|---|---|---|---|---|---|---|---|---|---|
| | 26 | NE | 29·77 | 29·76 | 29·765 | 58 | 47 | 52·33 | Fair |
| | 27 | NE | 29·82 | 29·74 | 29·295 | 58 | 48 | 53 | Fair |
| | 28 | NE | 29·90 | 29·82 | 29·867 | 56 | 46 | 52·33 | Fair |
| ○ | 29 | ENE | 30·02 | 30·— | 30·005 | 56 | 47 | 51·33 | Fair |
| | 30 | E | 30·— | 29·99 | 29·995 | 55 | 45 | 50·33 | Fair |
| | 1 | E | 30·02 | 30·02 | 30·02 | 55 | 39 | 47 | Fair |
| | 2 | E | 30·1 | 30·1 | 30·1 | 57 | 43 | 48·33 | Fair |
| | 3 | E | 30·22 | 30·18 | 30·196 | 57 | 40 | 47·66 | Fair |
| | 4 | E | 30·23 | 30·19 | 30·207 | 58 | 42 | 48·33 | Fair |
| | 5 | E by S | 30·07 | 29·95 | 29·997 | 59 | 39 | 49 | Fair |
| ◑ | 6 | SE | 29·85 | 29·72 | 29·767 | 59 | 39 | 46·33 | Fair during Day. Fog at Night. |
| | 7 | SW | 29·86 | 29·80 | 29·83 | 59 | 37 | 47·33 | Fair dur. Day. Fl. Show. p.m. Rain at Night |
| | 8 | W.WNW | 29·96 | 29·90 | 29·922 | 54 | 35 | 44 | Fair |
| | 9 | NNW | 30·13 | 30·— | 30·043 | 48 | 29 | 43 | Fair |
| | 10 | SE | 30·13 | 30·10 | 30·115 | 50 | 35 | 40·33 | Fair |
| | 11 | SW | 30·— | 29·84 | 29·982 | 54 | 45 | 49·33 | Fair |
| | 12 | W | 29·70 | 29·66 | 29·927 | 58 | 50 | 53 | Fair Cloudy |
| ● | 13 | S | 29·61 | 29·4 | 29·533 | 65 | 51 | 57·66 | Fair |
| | 14 | S | 29·47 | 29·4 | 29·43 | 65 | 48 | 60 | Cloudy Rain, p. m. |
| | 15 | S | 29·59 | 29·53 | 29·555 | 60 | 47 | 52·66 | Fair |
| | 16 | S by W | 29·7 | 29·6 | 29·645 | 57 | 42 | 49·66 | Fair Brisk Wind |
| | 17 | NW | 29·74 | 29·65 | 29·686 | 59 | 49 | 53·33 | Fair. Calm |
| | 18 | SW | 29·4 | 29·18 | 29·297 | 65 | 40 | 52 | Cloud. in Morn Heavy Rain pm & dur. Night |
| | 19 | SW | 29·22 | 29·2 | 29·213 | 54 | 42 | 48·66 | Rain |
| | 20 | SW | 29·44 | 29·39 | 29·41 | 52 | 40 | 45·66 | Cloudy |

RESULTS.

| | | | | | |
|---|---|---|---|---|---|
| Mean barometrical pressure 29·792 | | | Mean temperature | | 49·7 deg. |
| Maximum 30·23 | wind at | E | Maximum 65, | wind at | S |
| Minimum 29·2 | ——— | SW | Minimum 29, | ——— | SE |

*Scale exhibiting the prevailing Winds during the Month.*

| N | NW | W | SW | S | SE | E | NE |
|---|---|---|---|---|---|---|---|
| 1 | 1 | 2 | 5 | 4 | 2 | 6 | 4 |

| | Mean barometrical pressure. | Mean temperature. |
|---|---|---|
| From the full moon on the 29th Sept. to the last quarter on the 6th October | 30·074 | 48·854 |
| ——— last quarter on the 6th, to the new moon on the 13th | 29·94 | 46·188 |
| ——— new moon on the 13th, to the first quarter on the 21st | 29·471 | 52·453 |

*Observations.*

*Observations.*—October, 1814.—Greatest height of the mercurial column in the barometer, 30·23 inches on the 4th of October. Wind at E., the atmosphere clear, and the mean temperature of the 24 hours, 48·3 of Fahrenheit.

The lowest point of the mercurial column, 29·2 in. on the 19th. Wind at SW., the day and night rainy in smart showers, and the mean temperature of the 24 hours, 48·6 of Fahrenheit.

The highest temperature 65 on three several days, on the 13th and 14th with the wind at S., and on the 18th the wind SW. The highest mean temperature of the 24 hours is on the 14th, 60; the wind at S., and the mean barometrical height for the same period, 29 43 inches.

The lowest temperature 29, on the 9th; the wind NNW.; the atmosphere clear; the mean temperature of the 24 hours, 43; the mean barometrical height for the same period, 30 inches.

Wind N one day, NW one day, W two days, SW five days, S four days, SE two days, E six days, NE four days; the points of the wind being taken as they stood at eight, a. m.

Rain on four days, and during the night of those days; a considerable fog on the night of the 6th; the lowest temperature during that night, 39; the wind E by S.

From the full moon on the 29th September, to the last quarter on the 6th October, the mean barometrical pressure 30·07 inches; the mean temperature of that period, 48·85.

From the last quarter on the 6th, to the new moon on the 13th, the mean pressure, 29·94 inches; the mean temperature of the period, 46·18.

From the new moon on the 13th, to the first quarter on the 21st, the mean pressure, 29·47 inches; the mean temperature of that period, 52·45.

The mean barometrical pressure of this month's portion of the register, 29·792 inches; the mean temperature of the period, 49·7.

